# VEGF Polymorphisms Related to Higher Serum Levels of Protein Identify Patients with Hepatocellular Carcinoma

**DOI:** 10.1155/2016/9607054

**Published:** 2016-08-31

**Authors:** Maria Eduarda Lopes Baitello, Graciele Domitila Tenani, Rafael Fernandes Ferreira, Victor Nogueira, Marcela Augusta de Souza Pinhel, Rita de Cássia Martins Alves da Silva, Renato Ferreira da Silva, Patrícia da Silva Fucuta, Moacir Fernandes de Godoy, Dorotéia Rossi Silva Souza

**Affiliations:** ^1^Research Centre for Biochemistry and Molecular Biology, Medical School of São José do Rio Preto (FAMERP), São José do Rio Preto, SP, Brazil; ^2^Hepatology Unit, HB University Medical Centre, FUNFARME/FAMERP, São José do Rio Preto, SP, Brazil; ^3^Liver Transplantation Unit, HB University Medical Centre, FUNFARME/FAMERP, São José do Rio Preto, SP, Brazil

## Abstract

Hepatocellular carcinoma (HCC) is the most common primary neoplasia of the liver. Major risk factors for hepatocellular carcinoma include chronic liver diseases, carcinogenic agents, and genetic alterations as well as vascular endothelial growth factor (VEGF) involved in angiogenesis process. The aim of this study was to evaluate the association of* VEGF-A* (C936T and A1154G) with HCC and cirrhosis, in addition to serum levels of VEGF, clinical profile, lifestyle habits, and comorbidities. A total of 346 individuals were studied: 102 with HCC (G1), 117 with cirrhosis (G2), and 127 controls (G3). Polymorphisms were analysed by PCR/RFLP and serum levels of VEGF by ELISA. Alpha error was set at 5%. The wild-type genotype of both polymorphisms prevailed (*P* > 0.05). In G1, 23% of the patients died, with no relation to genetic profile (*P* > 0.05). Increased VEGF level was observed in G1 and G3, related to the mutant allele of* VEGF-*C936T and* VEGF-A*1154G, respectively, and compared with the wild-type genotype (*P* = 0.0285; *P* = 0.0284, resp.) as well as G1 versus G2 and G3 for* VEGF*-C936T and G1 versus G2 for* VEGF*-A1154G (*P* < 0.05 for both). In conclusion, there is a relationship between mutant alleles of* VEGF*-C936T and* VEGF*-A1154G polymorphisms and higher VEGF level, making them potential markers for HCC.

## 1. Introduction

Hepatocellular carcinoma (HCC) is the most common primary neoplasia of the liver, with increasing incidence and mortality [1]. In this context, emphasis should be given to Africa and especially to Asia, as China accounts for approximately 50% of world's HCC cases [2]. On the other hand, there is low incidence of the disease in North America and South America as well as in Europe [3]. High mortality is mainly due to the lack of follow-up on patients with chronic liver disease [4]. Imaging techniques, such as ultrasound, computed tomography, and magnetic resonance, are used for the diagnosis of the disease. In cases where X-ray exams have produced inconclusive results, histology is considered the gold standard [5].

HCC is a solid and well-vascularized malignant tumour, which usually develops from a chronic liver disease, being the cirrhosis responsible for 80–90% of cases. Hepatitis B virus (HBV) and C virus (HCV), alcohol consumption, smoking, and exposure to aflatoxins are also associated with an increased risk of developing the disease. Recently, the incidence of HCC has grown by hepatic steatosis [6–9].  Hepatocarcinogenesis can also result from the combination of genetic and epigenetic alterations in multiple signalling pathways, which affect cell proliferation, angiogenesis, cell invasion, and vascular permeability, leading to metastasis [10].

Neovascularization is an important mechanism in the pathogenesis of HCC because it contributes to the transition from dysplastic liver nodules into malignant phenotype [11]. In this context, the vascular endothelial growth factor-A (VEGF-A) has been reported as an important angiogenic factor in developing HCC [12]. The VEGF family consists of four* VEGF* genes (A–D) located in the human chromosome 6p21.3, which encode proteins that activate multiple signalling networks and promote endothelial cell growth, migration, differentiation, and control of vascular permeability [13, 14].

Studies show that* VEGF-A* polymorphisms are associated with an alteration in the promoter and 3′-UTR region of the gene, reflecting changes in plasma levels of the protein [15, 16].* VEGF*-C936T and* VEGF*-A114G were related to changes in plasma levels of the protein in several studies on solid tumours, but the results are controversial [17–19]. However, studies that correlate between polymorphisms of* VEGF* and HCC are rare, usually with small numbers of patients as well as a few polymorphisms [16, 20].

Moreover, within the limits of our knowledge, there are no studies in the literature involving polymorphisms of VEGF and HCC in Brazilian casuistic of interbred character, which contributed to the choice of these polymorphisms in this study.

Therefore, this study evaluated the association of* VEGF-A* polymorphisms (C936T and A1154G) with cirrhosis, HCC, and respective clinical classification, in addition to serum levels of VEGF and survival in HCC. It also considered risk factors for the disease, including comorbidities and lifestyle habits.

## 2. Methods

### 2.1. Materials

This case-control study included 346 individuals treated at the Hepatology Unit of Hospital de Base University Medical Centre (HB) at the Medical School of São José do Rio Preto (FAMERP). They were divided into three groups: G1, 102 patients with HCC; G2, 117 patients with cirrhosis; G3, 127 individuals without the disease (controls). The patients were classified according to the staging system set by* Barcelona Clinic Liver Cancer* (BCLC) in G1. Control subjects were selected at the Blood Centre at HB/FAMERP and were tested for blood-borne infections [21], including HBV and HCV. All participants were informed of the study and signed an Informed Consent Document. The project was approved by the Research Ethics Committee, CEP/FAMERP (protocol number: 6910/2011).

### 2.2. Genotyping

Genomic DNA was extracted from leukocytes of the total peripheral blood with EDTA using salting-out method [22]. The genotyping was performed by PCR/RFLP (polymerase chain reaction/restriction fragments length polymorphism) with a final volume of 25 *μ*L for the reactions as follows: 12.55 *μ*L of sterile MilliQ water, 2.5 *μ*L of DMSO (dimethyl sulfoxide), 2.5 *μ*L of 10x PCR buffer, 1.25 *μ*L of DNTP mix (10 mM) (deoxyribonucleotide triphosphate mix), 1.25 *μ*L of P1 primer (10 pmol/*μ*L), 1.25 *μ*L of P2 primer (10 pmol/*μ*L), 1.5 *μ*L of MgCl_2_ (25 mM), 1 U of Taq DNA polymerase (Thermo Scientific, Thermo Fisher Scientific, Inc., Massachusetts, USA), and 100 ng of DNA. PCR was performed under the following conditions: 5 minutes at 95°C, followed by 35 cycles at 95°C for 45 seconds, 61°C for 45 seconds, 72°C for 45 seconds, and finally at 72°C for 7 minutes. The primers used for genotyping were F5′-TAAATGTATGTATGTGGGTGGGTGTGTCACAGG-3′ and R5′-AAGGAAGAGGAGACTCTGCGCAGAGC-3′ for* VEGF*-C936T and F5′-TCCTGCTCCCTCCTCGCCAATG-3′ and R5′-GGCGGGGACAGGCGAGCATC-3′ for* VEGF*-A1154G. The amplification product was subjected to enzyme restriction with* Nla*III (*VEGF*-C936T) and* Mnl*I (*VEGF*-A1154G), followed by staining with GelRed (Uniscience, São Paulo, Brazil) and agarose gel electrophoresis of 2.5% and 4%, respectively. C-alleles (208 base pairs—bp) and T-alleles (122 bp and 86 bp) were identified for* VEGF*-C936T, and A-alleles (184 bp and 22 bp) and G-alleles (150 bp, 34 bp, and 22 bp) were identified for* VEGF*-A1154G.

Serum VEGF levels were obtained using ELISA (enzyme-linked immunosorbent assays, R & D System, Inc., Minneapolis, USA), in compliance with the manufacturer's instructions. For qualitative analysis, the reference value was set at 186.7 pg/mL, obtained by the cut-off value of the receiver operating characteristic curve (ROC).

The clinical classification of the patients was made based on the BCLC (A, B, C, or D) and Child-Pugh staging system (A, B, or C) according to the clinical practice guidelines of the American Association for the Study of Liver Diseases [5]. Patients classified as B and C of BCLC have been grouped due to the lack of portal invasion and/or metastasis of data from medical records of patients.

### 2.3. Statistical Analysis

The comparative analyses of allele frequencies and genotype distributions among the groups, as well as of further qualitative variables, were performed using the Chi-Square test with Yates correction or Fisher's exact test. Statistical models used for genetic profile analysis were dominant, recessive, and heterozygous. For the analysis of the Hardy-Weinberg equilibrium (HW), Chi-Square test was applied. Survival was analysed by Kaplan-Meier method and the results were compared by the log rank test. Quantitative variables were analysed using the Mann-Whitney test (within groups) and Kruskal-Wallis (between groups). For the evaluation of sensitivity, specificity, positive predictive value, and negative predictive value, the ROC curve was used, considering areas under the curve ≥0.7 as clinically relevant. A box-plot graphical representation was used, including minimum, interquartile range, median, and maximum values, as well as possible outliers. Alpha error was set at 5%. The programs used in the analyses were Minitab, Stats Direct, and GraphPad.

## 3. Results

Demographic profiles, lifestyle habits, comorbidities, and clinical classification of patients are shown in Table 1. Men accounted for 75% of subjects in all groups (*P* < 0.05). There was a higher frequency of alcohol in both groups of patients, G1 (56%) and G2 (50%), compared with controls (13%) (*P* < 0.0001 for G1 versus G3 and G2 versus G3) while G1 versus G2 did not show significance (*P* = 0.425). Yet, smoking prevailed in G1 (50%), compared with G2 (34%; *P* = 0.0257) and G3 (21%; *P* < 0.0001), while G2 versus G3 did not show significance (*P* = 0.0927). G1 showed higher frequency of cirrhosis (84%), followed by HCV (50%) and HBV (21%), and the same occurred for G2 (49% and 10%) with no significant difference between the groups (*P* = 0.957 and *P* = 0.052, resp.). The clinical classification of BCLC was performed in 89 patients in G1, with 31% belonging to the A classification, 59% to B or C, and 10% to D.

Table 2 shows the* VEGF-A* polymorphisms. The wild-type homozygous genotype for C936T (C/C) prevailed in G1 (71%), G2 (72%), and G3 (71%) as well as the wild-type allele (C = 0.85) in all groups. For the A1154G polymorphism, the wild-type genotype (G/G) also prevailed in all groups (60%, 51%, and 57%, resp.) as well as the wild-type allele (G = 0.77; 0.74; 0.76, resp.), with no significant difference between the groups (*P* > 0.05). HW equilibrium for both polymorphisms was observed in all groups (*P* > 0.05).

**Figure 1:**
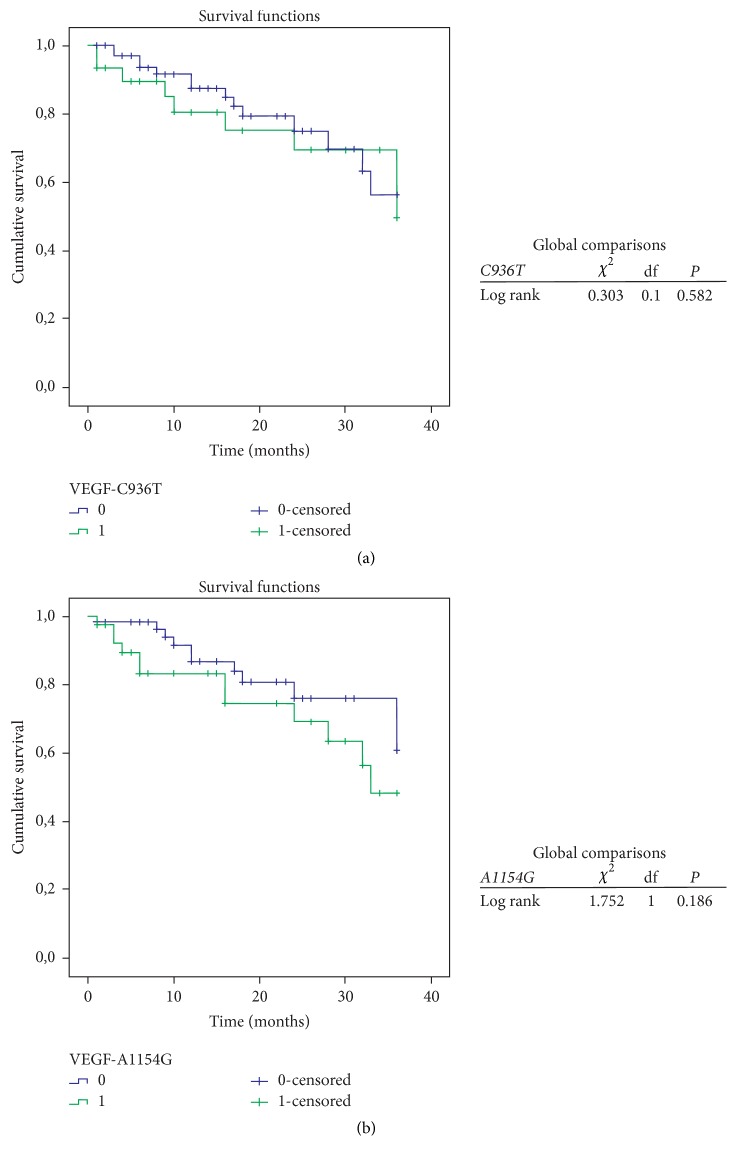
Kaplan-Meier curve for overall survival analysis of patients with hepatocellular carcinoma after 36 months of diagnosis. (a)* VEGF*-C936T: 1 = genotype C/T, 0 = genotype C/C; (b)* VEGF*-A1154G: 1 = genotypes _ /A, 0 = genotype G/G; df = degree of freedom.

Figure 1 shows the association between* VEGF-A* polymorphisms and survival of patients with HCC after 36 months of diagnosis. For* VEGF-*C936T, this study considered the heterozygous genotype (C/T) versus wild-type homozygous genotype (C/C), since homozygous mutant genotypes could not be found in the sample. Among patients, 72 (71%) had genotype C/C, of whom 14 (19%) died, with survival of 56.2 ± 10.9% in 36 months. For the C/T genotype, 9 of 30 patients (30%) died, with survival of 49.5 ± 13.8% in 36 months (*P* = 0.582). For* VEGF*-A1154G, the dominant statistical model (_ /A × G/G) was considered. Among 61 (60%) patients with G/G genotype, 11 (18%) died, corresponding to survival of 60.8 ± 11.3% in 36 months of follow-up. As for the genotype _ /A, 12 (29%) of 41 patients died, with survival rate of 48.3 ± 12.1% in 36 months (*P* = 0.186).

**Figure 2:**
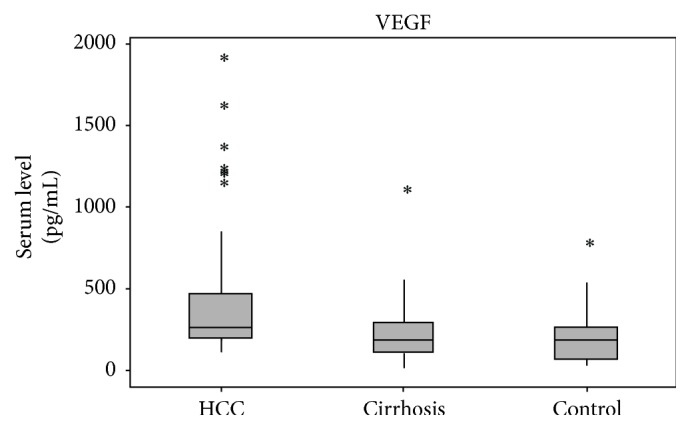
Box-plot representation of median and quartile values of serum vascular endothelial growth factor (VEGF) levels. HCC: median = 264.8, minimum = 102.0, maximum = 1795.1, Q1 = 199.4, Q3 = 467.7, and IQRange = 268.3;* cirrhosis:* median = 182.8, minimum = 7.2, maximum = 993.0, Q1 = 103.9, Q3 = 287.4, and IQRange = 183.5;* control*: median = 182.2, minimum = 31.6, maximum = 666.1, Q1 = 66.14, Q3 = 265.5, and IQRange = 199.4. ^*∗*^Extreme outliers of the distribution of serum levels values. Extreme outliers are observations that are beyond one of the outer fence OF1 or OF2. The outer fences are calculated as follows: OF1 = Q1 − 3*∗*IQR, OF2 = Q3 + 3*∗*IQR.

Table 3 shows the relationship between polymorphisms with BCLC clinical classifications in G1. The wild-type alleles and genotypes for both polymorphisms prevailed in all classifications, with no statistical difference (*P* > 0.05).  Increased serum levels of VEGF (Figure 2) were observed in G1 (264.8 pg/mL), compared with G2 and G3 (182.8 pg/mL; 182.2 pg/mL) (*P* = 0.0007 for G1 versus G2 and *P* = 0.0026 for G1 versus G3). Yet, serum levels of G2 versus G3 showed no significant differences. Serum VEGF levels were also analysed according to the genetic profile (Table 4). In HCC patients (G1), the elevated serum levels were related to the mutant allele (T) of* VEGF*-C936T (430.0 pg/mL), compared with the wild-type genotype (C/C) (250.5 pg/mL; *P* = 0.0285). G1 also showed elevated serum levels when compared to G2 (173.5 pg/mL) and G3 (113.9 pg/mL) (*P* = 0.0038) in an intergroup analysis with Kruskal-Wallis test. In relation to* VEGF*-A1154G, the serum levels of VEGF were increased in controls with the mutant allele (_ /A = 185.2 pg/mL) compared with genotype G/G (182.2 pg/mL; *P* = 0.0284). The comparative analysis also showed a significant increase in serum levels of VEGF in the presence of the mutant allele in G1 (297.8 pg/mL) compared to G2 (183.3 pg/mL; *P* = 0.0069), while G1 versus G3 and G2 versus G3 did not represent statistical differences.

**Figure 3:**
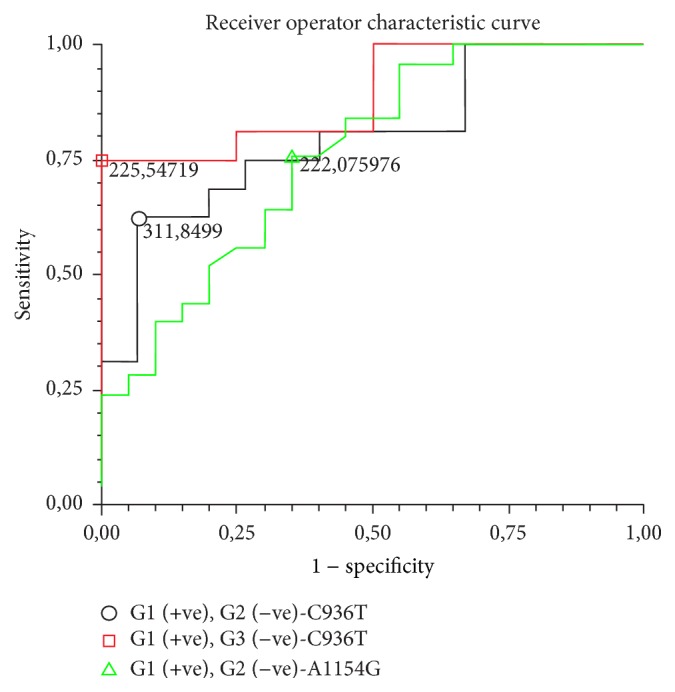
Receiver operator characteristic curve (ROC) of serum vascular endothelial growth factor (VEGF) levels in patients with (○) mutant allele of* VEGF*-C936T in the group with hepatocellular carcinoma (G1) and cirrhosis (G2) (area under the curve = 0.80 [0.60–1.0]), with sensitivity of 63% and specificity of 93%, to the cut-off value of 311.8 pg/mL; (□) mutant allele of* VEGF*-C936T in G1 and controls (G3) (area under the curve = 0.89 [0.60–1.0]), with sensitivity of 75% and specificity of 100%, to the cut-off value of 225.5 pg/mL; (∆) mutant allele of* VEGF*-A1154G in G1 and G2 (area under the curve = 0.76 [0.60–0.92]), with sensitivity of 76% and specificity of 65%, to the cut-off value of 222.1 pg/mL.

Predictive analysis of serum VEGF levels in the presence of the mutant allele was performed for both polymorphisms (Figure 3). For the* VEGF*-C936T, comparison of G1 versus G2 showed area under the curve of 0.80 (0.60–1.0), with 63% of sensitivity and 93% of specificity for cut-off of 311.8 pg/mL. The comparison of G1 versus G3 for the same polymorphism showed an area under the curve 0.89 (0.61–1.0), with 75% of sensitivity and 100% of specificity for the cut-off of 225.5 pg/mL.* VEGF*-A1154G analysis of G1 versus G2 showed an area under the curve of 0.76 (0.60–0.92), with sensitivity of 76% and specificity of 65% for the cut-off of 222.1 pg/mL.

## 4. Discussion

This study evaluated demographic profile, lifestyle, comorbidities, and clinical classification of patients with HCC and their association with* VEGF-A* polymorphisms, a key angiogenic factor in the development of HCC [12]. Men prevailed among patients, in agreement with global estimates (71%) [3]. Alcohol consumption prevailed among patients with HCC and those with cirrhosis, compared with controls. The association between chronic liver diseases and alcohol consumption is well established [23, 24]. Alcohol intake causes damage to the liver tissue, due to the action of endotoxins, oxidative stress, and inflammation, causing fibrosis in the hepatic tissue, which contributes to the development of cirrhosis and HCC [25]. There was a higher frequency of smoking among patients with HCC, in agreement with another study that compared such association with the exposure to mutagenic and carcinogenic components [26].

Prevalence of cirrhosis in HCC patients was also observed in this study, followed by HCV and HBV, consistent with an epidemiological study, whose frequencies varied between 80 and 90% for cirrhosis and 44 and 66% for HCV in HCC cases, but indicated the presence of HBV in 50% of patients [27]. The lowest HBV index in this study can be explained by increased access of this population to immunizations, compared with the world population, especially in Asia and Africa, where HBV infection is highly endemic [28].

The studied polymorphisms are located in the promoter region of* VEGF*-*A* (A1154G) and 3′-UTR (C936T). The 3′-UTR region of the gene contains key regulatory elements which are sensitive to hypoxia (condition that stimulates the expression of VEGF) and contributes to high variability in VEGF production among tissues [29]. Related to the promoter region of the gene, in vitro study showed that mutations in this region regulate the expression of the gene in peripheral blood mononuclear cells, suggesting that the regulation of VEGF expression occurs primarily at transcriptional level [30].

*VEGF*-C936T and* VEGF*-A1154G are polymorphisms located in an important angiogenesis signalling pathway related to the development and prognosis of HCC, a well-vascularized solid tumour which depends on neovascularization for its growth [31]. In this case, the relationship between the respective mutant alleles and decreased expression and plasma levels of VEGF has been reported [17, 18], which may be a protective factor against the development of HCC. However, this study did not find an association of* VEGF*-C936T as well as* VEGF*-A1154G with cirrhosis or HCC. There are reports of these polymorphisms in susceptibility to HCC in Italian and Chinese populations, which may also suggest a relationship between ethnicity and* VEGF* variants [16, 20].

Analysis of the survival curve considering* VEGF*-A1154G and* VEGF*-C936T polymorphisms was performed, showing decreased survival, although without significance, in the presence of the mutant allele for both polymorphisms. These findings agreed with another study that showed an association of the homozygous mutant genotype* VEGF*-A1154G with increased risk of death for HCC [32]. However, the small sample size may have been a limiting factor in the present study.

Although studies about the association between* VEGF* polymorphisms and survival in HCC are scarce, its influence on other types of cancer, such as colorectal and oesophagus, is recognised [33, 34]. Furthermore, the wild-type allele of* VEGF*-A1154G polymorphism was associated with poor survival in a Caucasian population with oral squamous cell carcinoma [35]. But the results are still diverging [36–39].  Increased serum levels of VEGF could only be observed in the HCC group, compared with the group with cirrhosis and controls, in agreement with a study by Mukozu et al. [40]. In this case, emphasis should be given to the relationship between VEGF and neovascularization and cell proliferation, both related to carcinogenesis, demonstrating the predictive value of VEGF for HCC. Analysis of serum VEGF levels according to the studied polymorphisms showed significant association between mutant alleles of both polymorphisms and increased serum VEGF levels, in agreement with a study, which showed the relationship between the mutant genotype of A1154G with poor prognosis in patients with HCC [41]. On the other hand, there are studies in which the mutant allele of* VEGF*-C936T was associated with decreased serum VEGF levels [15, 19].

Additionally, increased serum level of VEGF was observed in patients with HCC, particularly in the presence of the mutant alleles, compared with patients with cirrhosis and controls. This association highlights the action of the gene to promote vascularization and cell proliferation [42, 43] and strengthens the relationship between* VEGF* mutation and its serum level, contributing to the development of the disease. Serum levels of VEGF have been studied as potential markers for HCC. El-Sherif et al. reported a sensitivity of 60% and specificity of 92% for cut-off of 268.0 pg/mL [44]. El-Houseini et al. found 86.4% of sensitivity and 60% of specificity for the cut-off of 355.2 pg/mL [45]. In this study, the association of serum VEGF levels with the mutant alleles of* VEGF-A* (C936T and A1154G) showed cut-off value of 225.5 pg/mL, with specificity of 100% and sensitivity of 75% (C936T) comparing G1 and G3, conferring potential to serum VEGF levels in the diagnosis of HCC, which should be confirmed in numerous patient samples.

## 5. Conclusion

This study showed that* VEGF-A* polymorphisms (C936T and A1154G) are not associated with cirrhosis, HCC, and survival. However, there is a relationship between increased serum levels of VEGF and the presence of mutant alleles of both polymorphisms, which may contribute to the diagnosis and prognosis of HCC.

## Figures and Tables

**(a):** 

Variable	G1 (*N* = 102)		G2 (*N* = 117)		G3 (*N* = 127)		*P* ^*∗*^ value		
	*N*	(%)	*N*	(%)	*N*	(%)	G1 × G3	G2 × G3	G1 × G2
*Gender*									
Female	25	(25)	29	(25)	32	(25)	0.904	0.941	0.962
Male	77	(75)	88	(75)	95	(75)			

*Lifestyle*									
Alcohol consumption	57	(56)	58	(50)	18	(13)	**<0.0001 **	**<0.0001 **	0.42
Smoking	51	(50)	40	(34)	30	(21)	**<0.0001 **	0.0927	**0.0257**

*Comorbidity*									
HBV	21	(21)	12	(10)					0.0521
HCV	51	(50)	57	(49)					0.957
Cirrhosis	86	(84)	117	(100)					

**(b):** 

Clinical classification	G1 (*N* = 89)								
	*N*	(%)							
*BCLC*									
A	28	(31)							
B/C	52	(59)							
D	9	(10)							

^*∗*^Fisher's exact test or Chi-Square test; *N* = number of subjects; HBV = hepatitis B virus; HCV = hepatitis C virus; BCLC = Barcelona Clinic Liver Cancer.

**Table:** (a) C936T

	G1 (*N* = 102)		G2 (*N* = 117)		G3 (*N* = 217)		*P* ^*∗*^ value		
							G1 × G3	G2 × G3	G1 × G2
Allele	*n*	AF	*n*	AF	*n*	AF			

C	174	0.85	199	0.85	217	0.85	0.966	0.903	0.941
T	30	0.15	35	0.15	37	0.15			

Genotype	*N*	(%)	*N*	(%)	*N*	(%)			

Dominant									
C/C	72	(71)	84	(72)	90	(71)	0.963	0.985	0.962
C/T + T/T	30	(29)	33	(28)	37	(29)			
Heterozygote									
C/T	30	(29)	31	(26)	37	(29)	0.963	0.751	0.742
C/C + T/T	72	(71)	86	(74)	90	(71)			
Recessive									
T/T	—	(0)	2	(2)	—	(0)	—	—	—
C/T + C/C	102	(100)	115	(98)	127	(100)			
HW (*χ* ^2^)	3.03		0.20		3.69		>0.05		

**Table:** (b) A1154G

	G1 (*N* = 102)		G2 (*N* = 117)		G3 (*N* = 217)		*P* ^*∗*^ value		
							G1 × G3	G2 × G3	G1 × G2
Allele	*n*	AF	*n*	AF	*n*	AF			

G	157	0.77	172	0.74	193	0.76	0.893	0.598	0.469
A	47	0.23	62	0.26	61	0.24			

Genotype	*N*	(%)	*N*	(%)	*N*	(%)			

Dominant									
G/G	61	(60)	60	(51)	73	(57)	0.826	0.399	0.258
G/A + A/A	41	(40)	57	(49)	54	(43)			
Heterozygote									
G/A	35	(34)	52	(44)	47	(37)	0.776	0.293	0.164
A/A + G/G	67	(66)	65	(56)	80	(63)			
Recessive									
A/A	6	(6)	5	(4)	7	(6)	0.904	0.880	0.815
G/A + G/G	96	(94)	112	(96)	120	(94)			
HW (*χ* ^2^)	0.74		2.32		0.02		>0.05		

^*∗*^Fisher's exact test or Chi-Square test (*χ* ^2^); *N* = number of subjects; *n* = number of alleles; AF = absolute frequency; HW = Hardy-Weinberg.

**Table:** (a) C936T

*VEGF*	BCLC: 89 patients						*P* ^*∗*^ value		
	A (*N* = 28)		B/C (*N* = 52)		D (*N* = 9)		A × B/C	A × D	B/C × D
Allele	*n*	AF	*n*	AF	*n*	AF			

C	48	0.86	88	0.85	16	0.89	1.000	1.000	1.000
T	8	0.14	16	0.15	2	0.11			

Genotype	*N*	(%)	*N*	(%)	*N*	(%)			

C/C	20	(71)	36	(69)	7	(78)	1.000	1.000	0.7131
C/T	8	(29)	16	(31)	2	(22)			
T/T	0	(0)	0	(0)	0	(0)	—	—	—

**Table:** (b) A1154

*VEGF*	BCLC: 89 patients						*P* ^*∗*^ value		
	A (*N* = 28)		B/C (*N* = 52)		D (*N* = 9)		A × B/C	A × D	B/C × D
Allele	*n*	AF	*n*	AF	*n*	AF			

G	41	0.73	83	0.80	14	0.78	0.4508	1.000	0.7623
A	15	0.27	21	0.20	4	0.22			

Genotype	*N*	(%)	*N*	(%)	*N*	(%)			

G/G	14	(50)	35	(67)	5	(56)	0.2023	1.000	0.7056
A/G	13	(46)	13	(25)	4	(44)	0.0888	1.000	0.2489
A/A	1	(4)	4	(8)	0	(0)	0.6525	—	—

^*∗*^Fisher's exact test or Chi-Square test with Yates correction; BCLC = Barcelona Clinic Liver Cancer; VEGF = vascular endothelial growth factor; *N* = number of subjects; *n* = number of alleles; AF = absolute frequency.

**Table:** (a) *VEGF*-C936T

	VEGF serum levels × polymorphisms			Intergroups *P* ^*∗*^ value G1 × G2 × G3
	G1 (*N* = 54)	G2 (*N* = 51)	G3 (*N* = 27)	
	Median (pg/mL) (Minimum–maximum)			
C/C	250.5 (*N* = 38) (101.9–1120.2)	191.7 (*N* = 36) (7.23–992.95)	185.7 (*N* = 23) (41.4–666.1)	0.0700
_ /T	430.0 (*N* = 16)^▲■^ (133.35–1795.1)	173.5 (*N* = 15)^▲^ (50.1–556.2)	113.9 (*N* = 4)^■^ (31.56–225.45)	**0.0038**
*Intragroup* *P* ^*∗*^ * value*	**0.0285**	0.2868	0.0955	

**Table:** (b) *VEGF*-A1154G

	VEGF serum levels × polymorphisms			Intergroups *P* ^*∗*^ value G1 × G2 × G3
	G1 (*N* = 54)	G2 (*N* = 51)	G3 (*N* = 27)	
	Median (pg/mL) (Minimum–maximum)			
G/G	238.2 (*N* = 29) (101.9–1795.1)	182.8 (*N* = 31) (50.1–992.95)	182.2 (*N* = 17) (31.56–666.1)	0.0644
_ /A	297.75 (*N* = 25)^●^ (104.25–1256.3)	183.3 (*N* = 20)^●^ (7.23–556.17)	185.2 (*N* = 10) (41.4–534.0)	**0.0069**
*Intragroup* *P* ^*∗*^ * value*	0.18	0.1602	**0.0284**	

^*∗*^Mann-Whitney and Kruskal-Wallis tests for intragroup and intergroup analyses, respectively; VEGF = vascular endothelial growth factor; *N* = number of subjects; ▲ and ■: *P* < 0.05; ●: *P* < 0.01.
